# Application of single-cell sequencing to the research of tumor microenvironment

**DOI:** 10.3389/fimmu.2023.1285540

**Published:** 2023-10-27

**Authors:** Sijie Chen, Zhiqing Zhou, Yu Li, Yuhui Du, Guoan Chen

**Affiliations:** Department of Human Cell Biology and Genetics, Joint Laboratory of Guangdong-Hong Kong Universities for Vascular Homeostasis and Diseases, School of Medicine, Southern University of Science and Technology, Shenzhen, China

**Keywords:** single-cell sequencing, tumor microenvironment, clinical applications, precision medicine, immunological therapy

## Abstract

Single-cell sequencing is a technique for detecting and analyzing genomes, transcriptomes, and epigenomes at the single-cell level, which can detect cellular heterogeneity lost in conventional sequencing hybrid samples, and it has revolutionized our understanding of the genetic heterogeneity and complexity of tumor progression. Moreover, the tumor microenvironment (TME) plays a crucial role in the formation, development and response to treatment of tumors. The application of single-cell sequencing has ushered in a new age for the TME analysis, revealing not only the blueprint of the pan-cancer immune microenvironment, but also the heterogeneity and differentiation routes of immune cells, as well as predicting tumor prognosis. Thus, the combination of single-cell sequencing and the TME analysis provides a unique opportunity to unravel the molecular mechanisms underlying tumor development and progression. In this review, we summarize the recent advances in single-cell sequencing and the TME analysis, highlighting their potential applications in cancer research and clinical translation.

## 1 Introduction

**Figure 1:**
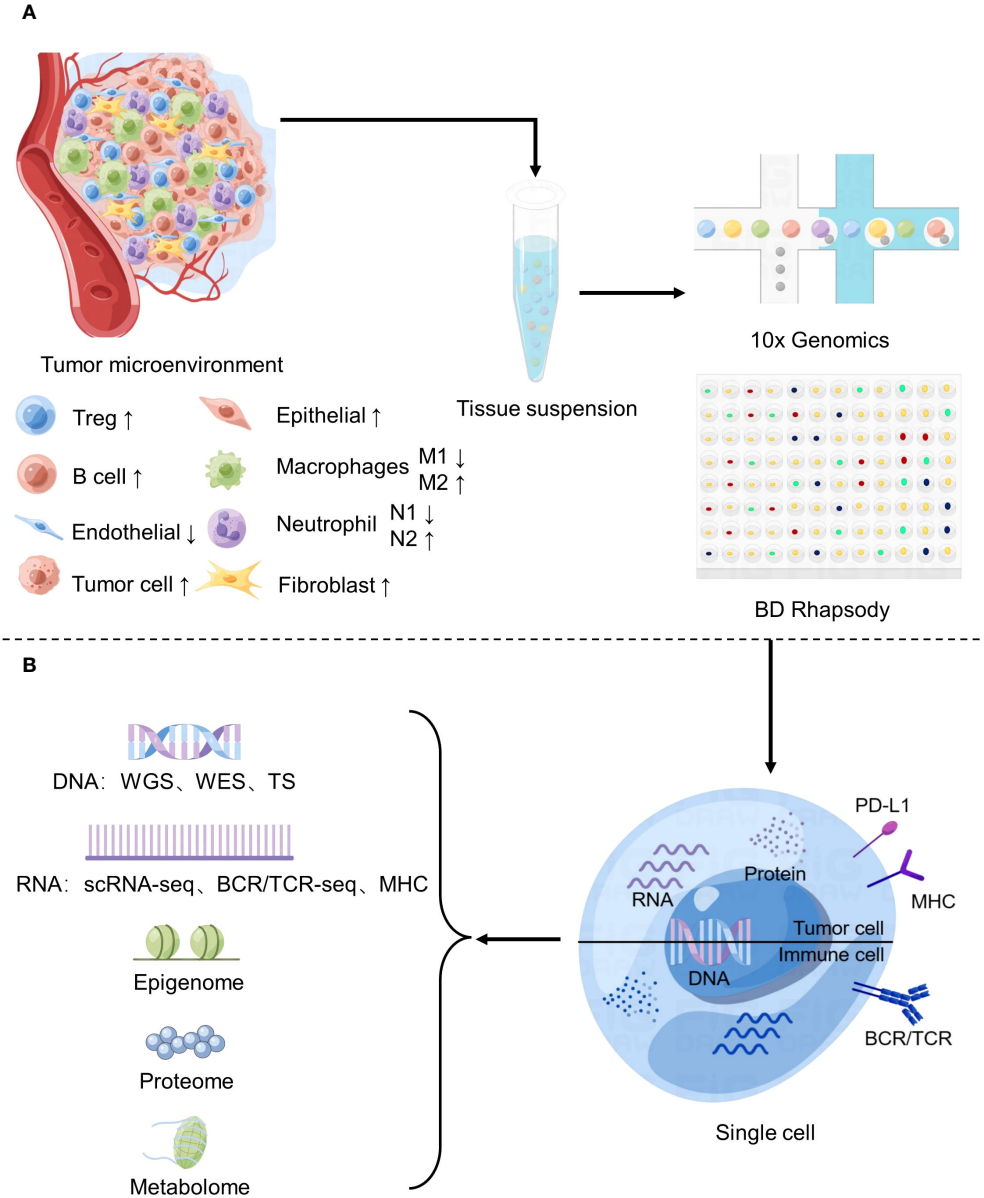
Application of Single-cell sequencing in the study of tumor microenvironment. **(A)** Changes of major cell components of tumor microenvironment and basic flow of single cell sequencing. **(B)** Common single-cell sequencing techniques.

Cancer is a complex disease characterized by genetic and epigenetic alterations that alter cellular processes (1), such as cell proliferation, cell death and angiogenesis (2). The current paradigms of cancer therapies are based on targeting specific signaling pathways that drive mutations or disorders. In contrast to the undifferentiated cytotoxicity of conventional standard chemotherapy, targeted therapies can interact with genes or proteins specific to tumor cells without killing normal cells (3). However, cells within a tumor still exhibit significant heterogeneity, which means different cloning patterns can coexist within a tumor, leading to treatment resistance and recurrence (4). For example, somatic loss of PTEN expression has been observed in patients with metastatic breast cancer treated with long-term phosphatidylinositol-3-kinase (PI3K) inhibitors, leading to therapeutic resistance (5).The main reason is that mutations in tumor driver genes offer a growth or survival advantage to these cells, they multiply in enormous numbers to become dominant clones of primary malignancies (6), however, there is still a certain degree of subclone type within tumors because of the high mutation of tumor genes (7), when driver genes no longer provide a selection advantage, other subclones assume their place (8), Thus reducing the inhibitory effect of drugs on their own growth. However single-cell sequencing technology enables the identification and characterization of individual cancer cells’ genomic and transcriptomic profiles (**Figure 1**), providing a more detailed understanding of intra-tumor heterogeneity (9), while eradicating the main clonal cell population, it may continue to precisely attack the subclone cell region and build a combination treatment plan for tumor cells and TME, which can more effectively enhance patient survival rates.

The TME is the immediate environment for survival of tumor cells (10), and is composed of various cell types and their metabolites. The main components include immune cells, stromal cells, endothelial cells, and extracellular matrix components (**Figure 1**). These complex components constitute a variety of structures, each playing a different role in tumor progression. such as blood vessels that provide oxygen and nutrients and remove metabolic waste (11), and signaling molecules secreted by stromal cells and immune cells that make up the extracellular matrix (ECM) to regulate tumor growth (12). So, the TME not only provides structural support to the tumor but also modulates its behavior by secreting cytokines, growth factors, and other signaling molecules. The TME plays a critical role in tumorigenesis, metastasis, and response to therapy. In particular, some studies have shown that stromal cells and cytokines play a role in inhibiting tumor growth in the early stage of tumor formation, but as time goes by, they slowly show to promote tumor growth and develop therapeutic resistance (13). Therefore, analyzing the TME’s composition, interactions, and functions is essential to unravel the complex interplay between cancer cells and their surrounding environment.

As a result, using single-cell sequencing technology to examine the composition, interaction and function of the TME to reveal the complex interaction between cancer cells and their surrounding environment will be the key direction of tumor microenvironment research in the next few years. Here we review common single-cell sequencing technologies, discuss the most recent research progress of each component of the tumor microenvironment, analyze the existing technical shortcomings of single-cell sequencing technology, and emphasize the application and prospect of single-cell sequencing technology in the analysis of various tumor microenvironments.

## 2 Single-cell sequencing technologies

Traditional bulk sequencing methods provide an average view of genomic and transcriptomic profiles, which can mask rare or low-abundance subpopulations, resulting in incomplete understanding of intra-tumor heterogeneity (14). Before single-cell sequencing technology was developed, flow cytometry was also tried to analyze individual cells from tumor tissues. However, this technique was still limited to analyze only a few specific immune cells, ignoring the contribution of other stromal cells and some undefined cell groups to tumor growth (15). Single-cell sequencing allows the profiling of individual cells, providing a more precise view of genetic and transcriptional diversity within a tumor. Since it was first reported in 2009 (16), single-cell sequencing technology has opened a new avenue to reveal the potential cellular heterogeneity of complex systems (17).

**Figure 2:**
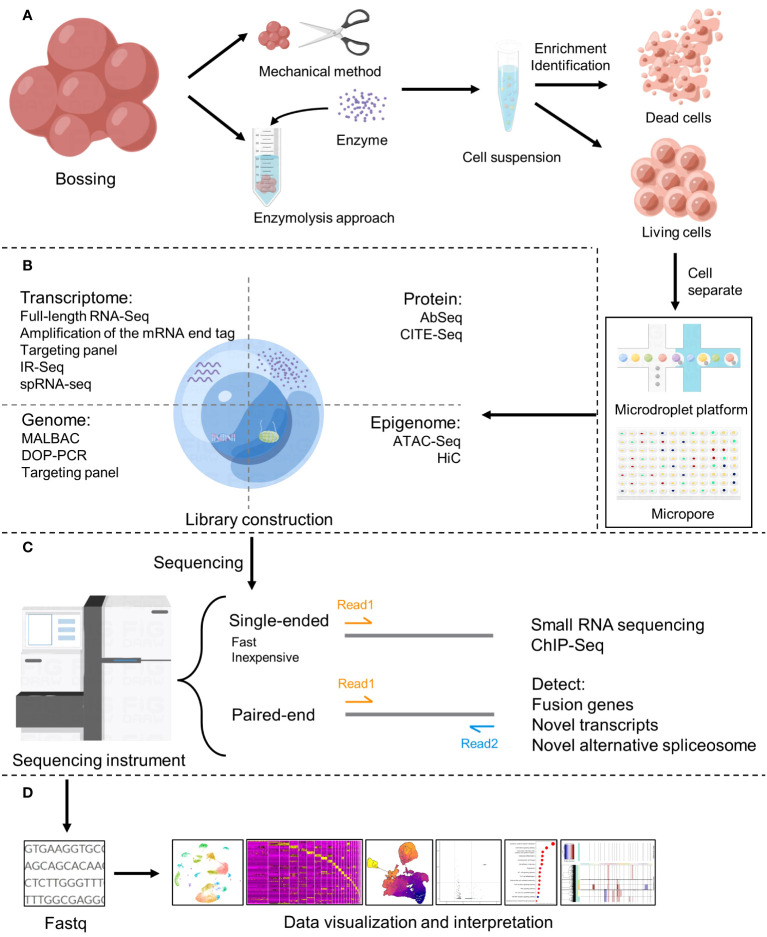
Basic flow of single-cell sequencing. **(A)** Single-cell preparation and isolation (18). **(B)** Library construction (19). **(C)** Sequencing (20). **(D)** Data analysis, visualization and interpretation.

The workflow of single cell sequencing consists of four main steps, including single-cell preparation and isolation, library construction, sequencing and primary analysis, data visualization and interpretation, and each step is crucial in the entire workflow, while the core of the whole step is the differentiation of matched tissues into individual cells (**Figure 2**). After separating a single cell, the chemicals within the cell need to be catch, and single-cell sequencing technologies have two main categories: DNA-based and RNA-based methods (21) (**Figure 1B**).

### 2.1 DNA-based methods: single-cell genome sequencing

#### 2.1.1 Introduction to single-cell genome sequencing

DNA-based methods which include whole-genome sequencing (WGS), whole-exome sequencing (WES) (22), and targeted sequencing (TS) is characterized by three core capabilities, which we term Fidelity, Co-presence, and Phenotypic association (23).

WGS involves the fragmentation and sequencing of an entire genome without preselecting specific DNA sequences. Conventional WGS can infer the overall mutation process of a tumor. But the mutations caused by genomic instability between cells after mitosis cannot be detected due to the muti-source signal collection from a huge number of different cells (24). Single-cell WGS can overcome this shortcoming. single-cell WGS can easily separate clonal specificity from cell genome time, and can calculate the cumulative rate and mutation pattern of copy number variation (CNV) and structural variation (SV) in a single-cell (25). However, WGS is still expensive and has limited accuracy for detecting low-frequency variants.

WES is a high-throughput genomics approach that uses probes or primers to target the exonic sections of the genome of interest, which contains the bulk of protein-coding genes. The collected DNA fragments are then submitted to high-throughput sequencing, and the exon sequence is determined by aligning them with the reference genome (26). Since exons make up 2% of the entire genome, WES is a more cost-effective approach with lower capital cost and time than WGS (27), which make it a potential powerful clinical diagnostic tool for the genetic basis of diseases. However, due to uneven sequence reading coverage on exome targets in the detection process, many low coverage areas appear. Besides, current studies show that non-coding regions still regulate genes, so WES is still limited in terms of non-coding variation and intron mutation affecting gene expression (28).

TS uses PCR or hybridization-based methods to capture and enrich the genomic region of interest and then conduct high-throughput sequencing (29). It can monitor the genetic variation sites of the target genomic region and obtain the variation information of the specified target region, which makes the detection of mutations of cancer-related genes with high sensitivity and specificity. Compared with WGS and WES, it provides deeper coverage and higher data accuracy to the target area, shortens the research period, and reduces the sequencing cost, and is suitable for the study of a huge number of samples (30).

#### 2.1.2 Application of single cell genome sequencing in cancer research

Single-cell genome sequencing has mostly been used in cancer research to investigate intra-tumor heterogeneity, clonal evolution, invasion and metastasis, circulating tumor cells, and therapy response (23). Tumor mutation burden(TMB) and alterations in karyotype structure, as well as the variety of tumor carcinogenic content and immunogenicity, all contribute to intra tumoral heterogeneity (ITH) (31). While ITH can be inferred from bulk DNA sequencing data obtained from conventional genome sequencing (32), single-cell genome sequencing can provide a more comprehensive, high-resolution view of ITH and clonal evolution (32). The 2010 work by Navin N et al. (33) was the first to describe ITH using single-cell genome sequencing technologies. They used the results of single-cell genome sequencing to CNV in breast cancers to differentiate subclonal lineages, indicating that the proliferation of subclones within tumors is the seed of metastasis. Following that, single-cell genomic techniques were used to investigate CNV and point mutations in a variety of solid tumors (34–40) and non-solid tumors (41–43) and it was discovered that most tumors contained more than one major subclonal lineage and a large number of low-frequency subclonal lineages, and subclonal diversity was discovered to be a marker of poor prognosis (44). Single-cell genome sequencing technology can also be used to detect the presence of cancer, and a large number of studies (33, 36, 39, 45, 46) have discovered that high-impact genomic point mutations and mutations in specific driver genes are the cause of cancer using this technology, contradicting the previously held belief that cancer is caused by the accumulation of point mutations (47). Furthermore, earlier tumor diagnosis was usually done by microscopy following puncture sampling, which might not only hurt the patient but also promote fake tumor spread due to tumor cell shedding during surgery. With the advent of single-cell genome sequencing, noninvasive tumor sampling methods for circulating tumor cells (CTC) have been developed, which can detect not only the entire tumor cell genome, but also early clonal mutations shared by both primary and metastatic cells (48), which can inform treatment (49).

#### 2.1.3 Advantages and disadvantages of single-cell genome sequencing

The heterogeneity (50) of the cell population genome that may be discovered and the small sample size required are two clear benefits of single-cell genome sequencing over traditional genome sequencing. Because there are many subclone cell populations that are not dominant in the tumor (51), conventional genome sequencing can only detect cell populations with obvious survival sites and cannot target subclone cell populations, which may be a contributing factor to tumor recurrence, metastasis, and drug resistance (52). The use of single-cell genome sequencing technology may concurrently target a number of clonotype cells for combination therapeutic usage, allowing for more accurate hits and a lower risk of patient recurrence, metastasis, and drug resistance (53). For conventional genome sequencing, current third-generation sequencing technologies require sample sizes greater than or equal to 5ug or proposed DNA concentrations greater than or equal to 30ng/ul (54), which is difficult to achieve for some precious and rare samples, such as circulating tumor cells (55). Multiple Displacement amplification (MDA) or multiple annealing and Multiple Annealing and Looping-Based Amplification Cycles (MALBAC) are often employed for genome amplification in single-cell genome sequencing (56), lowering the necessity on sample quality.

However, since the steps of genome amplification have been integrated into the library of single-cell genome sequencing, the question of whether the genome can overcome the problems of base mismatch, bias, coverage, allelic detachment, and so on, i.e., whether the amplification is uniform and effective, has become a key limiting the development of this technology. MDA, for example, is a popular amplification technique that may give quick and efficient amplification. Amplification with a high coverage power but a bias for certain sequences will result in uneven amplification. MALBAC can offer uniform amplification, but its coverage is limited, therefore it is frequently utilized to detect CNV (57). Because the current amplification techniques cannot fulfill the requirements of large coverage and high uniformity at the same time, the particular selection of amplification methods must be made based on the experimental goal.

In conclusion, even if single-cell genome sequencing technology has technological limits, individuals will continue to upgrade it, broaden its application area, and aid in the investigation of the occurrence and progression of cancer.

### 2.2 RNA-based methods: single-cell transcriptome sequencing

RNA-based methods include single-cell RNA-sequencing (scRNA-seq), single-cell TCR/BCR sequencing, and spatial RNA sequencing derived from scRNA-seq (spRNA-seq). scRNA-seq allows the profiling of the transcriptome at single-cell resolution, enabling the identification of cell types, gene expression patterns, and signaling pathways involved in cancer development (58). scRNA-seq can be performed using different methods, including SMART-seq, Drop-seq, and 10x Genomics (59). TCR/BCR sequencing enables the profiling of the immune repertoire at single-cell resolution, providing insights into the clonality and diversity of T and B cells (60).

#### 2.2.1 Introduction to single-cell transcriptome sequencing

Since the first EST library was sequenced in 2007 using a Roche 454 sequencer (61), Bulk RNAseq was once the most valuable and widely used tool for understanding cancer biology (62). In the past, single short-ended sequencing in Bulk RNAseq was commonly used to focus on differentially expressed genes to understand the molecular mechanism of various stages of tumorigenesis (63). Bulk RNAseq is most widely used in cancer diagnosis, prognosis, and predictive biomarkers. However, in fact, it is still poor in predicting prognosis of tumor patients, and one of the main reasons is inherent sampling bias caused by intra-tumor heterogeneity (64).

Therefore, after the emergence of scRNA-Seq in 2009, scRNA-Seq has become the best method to reveal the heterogeneity and complexity of RNA transcriptomes within a single-cell, as well as the different cell types and functional compositions in tissues and organs (58). The scRNA-seq has the advantage of being able to analyze both coding and non-coding transcripts, allowing the identification of novel gene isoforms, alternative splicing events, and non-coding RNAs that might have functional roles in carcinogenesis. Until now, single-cell sequencing has been focused on RNA-Seq (62). The current method used for conducting scRNA-seq can be used for Smart-seq2 and CEL-Seq2 at low throughput, while 10x Chromium has the best effect when dealing with high throughput (59). However, scRNA-seq has several technical challenges, including low capture efficiency, high technical noise, and batch effects. Therefore, careful experimental design, quality control, and data normalization are critical for accurate interpretation of scRNA-seq data. Even now, there is a relatively complete system for the analysis of scRNA-seq data, which can help researchers better mine information from clinical data (58).

Lundeberg, a Swedish scientist, presented the notion of spatial transcriptome in 2016 (65), a revolutionary new technology for RNA sequencing has emerged, namely spatial RNA sequencing (spRNA-seq), which combines whole-transcriptome analysis with *in situ* hybridization. The spRNA-seq provides spatial information for the entire transcriptome data, helping researchers further study how tumor cells communicate with each other, evade immune surveillance, and develop drug resistance. It provides a more effective treatment strategy for tumor patients (66) The current method used for conducting spRNA-seq can be used for the 10X spatial transcriptomics from 10X Genomics and digital spatial profiler(DSP) from Nanostring Technologies. Due to technological difficulties, few scientists had researched single-cell spatial transcriptomes before to the development of 10X spatial transcriptomics, and it was not until the appearance of 10X spatial transcriptomics that single-cell spatial transcriptome-related studies progressively became popular. The subsequent DSP offers various advantages over 10X spatial transcriptomics, including cost reductions, removing highly expressed molecules with no actual information, and tailoring targets of interest to quantify unique transcript variations (62). In summary, spRNA-seq adds spatial information to the entire transcriptome data, allowing us to better understand the highly structured spatial and temporal communication patterns between the various cells that comprise the tumor (66) and thus, it is essential for cancer diagnosis, subtyping, classification, and treatment (67), facilitating personalized, precisely targeted therapy for patients, and improving patient survival (68).

T and B cells are the adaptive immune system’s primary effector cells. Lymphocytes with various surface antigens will create diverse functions during tumor growth, implying that different TCR/BCR can take on particular roles (69). However, it is widely known that their manufacturing process will involve a number of combinations, resulting in a complicated and diversified TCR/BCR (70). Different TCR/BCR libraries should be evaluated in order to comprehend the individual roles of T cells and B cells in tumor occurrence and progression, and then the technique of integrating TCR/BCR sequencing with single-cell sequencing has evolved. The introduction of this technology has accelerated the growth of the field of T cells, allowing researchers to better understand the function and physiological significance of T cells and B cells in immune response by studying TCR libraries with cell state information. At the moment, people may extract TCR/BCR information from RNA-SEQ data by combining the method with comparison and reassembly, and then the TCR/BCR chain can be rebuilt (71). For example, ElhamAzizi’s article in 2018 revealed the complex tumor immune microenvironment through the sequencing of the single-cell immune set library, and discovered that TCR diversity was not the driving factor for the continuous activation of T cells, but that there were other factors, further understanding the molecular mechanism behind the promotion and delay of tumor immune cell growth (72). It can be seen that the application of single-cell sequencing in immunologic is designed to understand the dynamics of immune cell cloning in tissues and *in vitro* by determining the clonal type of immune cells (72), identify the composition and diversity of immune group library sequences, explore gene expression, discover new biomarkers, and analyze clonal differences within tumors or between tumors and adjacent tissues (73).

#### 2.2.2 Application of single cell transcriptome sequencing in cancer research

Heterogeneity within tumors is produced not only by genomic abnormalities, but also by molecular aberrations at the transcriptome level (74), because of variations in transcriptome activity and regulation constitute the foundation of cell phenotypic variety, To examine the transcriptome at the single-cell level, single-cell transcriptome sequencing technique is applied (75). Provides the ability to identify and define transcriptionally different subpopulations and states that may impact clinical outcomes, guide treatment tactics, or hint to novel therapy options (76). After years of development, downstream data analysis essentially has a set of patternized flow (**Figure 2D**). Other personalized analysis flow, such as the calculation of allele expression to identify single nucleotide variation (SNV) or CNV, the analysis of evolutionary trajectory to characterize transcriptional dynamics, splicing detection from coding receptor and ligand gene expression and so on (74), that can be selected according to the purpose of analysis. At the moment, single-cell transcriptome sequencing technique is frequently employed in cancer research, with excellent results. In 2021, Jeffrey J et al. (77) used CRISPR-Cas9-based single-cell transcriptome analysis to map detailed malignant cell profiles in a mouse model of KRAS mutation, demonstrating that single-cell transcriptome sequencing technology overcomes the challenge of low mutation sensitivity and the inability to decipher the changing details of tumor subtypes. This aids in the tracking of cancer spread patterns and important genes, helping in the development of targeted medicines and the improvement of clinical care techniques for patients with KRAS mutations. Since the PD-1/PD-L1 pathway was awarded the Nobel Prize in Physiology and Medicine in 2018 (78), PD-L1 is the first biomarker approved as a companion test to the prescription of an immune checkpoint inhibitor targeting PD-1. However, PD-L1 remains an unsatisfactory biomarker due to a high false positive or false negative rate during routine testing (79), and the identification of additional immune detection sites suffers from the same issue (80, 81). Incorrect identification of immunological checkpoints leads to incorrect therapy, and the number of patients who benefit long-term is restricted (82, 83). However, scRNA-seq can improve the accuracy of detection, which can solve this problem. For example, patients with highly immunogenic tumors with high PD-L1 expression and CD8+T cell and dendritic cell infiltration, have been observed to respond to immune checkpoint blockers more than patients with non-immunogenic tumors, and such patients may benefit from immune checkpoint blockers in their long-term treatment (84). Currently, in clinical practice, there are research on the changes in tumor immune cell population rate in response to various therapies using scRNA-seq for patients, in order to alter medicine (85), and ultimately achieve the goal of prolonging patient survival.

#### 2.2.3 Advantages and disadvantages of single-cell transcriptome sequencing

Single-cell transcriptome sequencing technology provides higher resolution for gene expression studies than traditional transcriptome sequencing technology because cells differ from one another in multicellular organisms, which may be reflected in different genetic backgrounds, different differentiation states, and heterogeneity. Various physical traits, gene mutation profiles and transcriptome, proteome expression patterns, and so forth (86). Due to the tumor’s heterogeneity, distinct subgroups within the tumor exhibit varied immunological features, growth rate, and capacity to metastasize, as well as varying clinical responses to treatments. Single-cell transcriptome sequencing technique, on the other hand, generates the expression profile of each cell at the level of a single cell, considerably enhancing the resolution of cell diversity research. This is primarily represented in the ability to categorize cells based on gene expression, and then do specific differential expression analysis of cell populations to identify critical genes associated to tumor formation and undertake targeted medication guiding (15, 76). Because temporal and spatial variability affect tumor heterogeneity and stress response, however single-cell spatial transcriptome sequencing adds the latitude of time and space to the tumor transcriptome map, facilitating the study of the local environment and dynamic interactions within a single cell (67, 87).

Despite its widespread use and rapid growth, single-cell t ranscriptome sequencing has several drawbacks. For instance, there are numerous platforms (75) based on data base set-up, with significant disparities in coverage, geographical bias, and database construction cost. For example, despite the fact that the most common 10Genomics technology on the market is less expensive and can detect more cells at the same time, RNA amplification is restricted to a few dozen pb at the 3’segment, disregarding changes induced by post-transcriptional modification (88). On the other hand, the Smart-Seq technology, stretches RNA to virtually the whole length, but at a high cost and with a limited number of cell recognitions (89). Furthermore, various platforms have variable capturing capacities for distinct cell subpopulations (90), which may result in the omission of a cell subpopulation. The second is data generated by various platforms that must be corrected before being compared, and the correction procedure may also disregard minor but significant variations. Other issue is that various persons have different nomenclature for the same set of cell subgroups with the same function due to variances in sequencing platforms and post-analysis. As well as the practical clinical application of single-cell spatial transcriptome sequencing, it is impossible to analyze single-cell spatial transcriptome by *in situ* sequencing, fluorescence *in situ* hybridization, *in situ* capture technology, and computer methods because samples are usually retained in formalin or paraffin for a long time. So, there is an urgent need to build a low-cost platform capable of analyzing formalin-fixed or paraffin-embedded tissues for clinical use.

In brief, single-cell transcriptome sequencing paired with spatial transcriptomics will considerably aid in the discovery of previously unknown biomarkers and medications implicated in tumor formation, paving the door for improved treatment results.

### 2.3 Prospect of single-cell sequencing technology

In addition to DNA-based methods and RNA-based methods, there are a number of other single-cell sequencing techniques, such as single-cell CRISPR screening technologies, SC epigenetics, SC proteomics methods, SC multi-omics technologies, Emerging SC technologies and methods and spatially resolved omics approaches. In conclusion, single-cell sequencing technology can significantly improve the understanding of disease, especially in the process of tumor research, broadly explain the diversity of cells within a tumor, track the heterogeneity of cancer cells, and map the immune niche (ecosystem or ecotype) of a matrix with unique cell composition (72).

Prior to the advent of single-cell sequencing, the understanding of tumor microenvironment was mainly analyzed and studied by histological and immunohistochemical techniques. This approach provides only general information about the different cell types in the entire tissue and does not allow for in-depth study of the subpopulations within them. The advent of single-cell sequencing technology allows scientists to better understand the composition and changes of the tumor microenvironment. Through single-cell sequencing, gene expression and functional status of different cell types and their subpopulations can be accurately isolated, analyzed and described. This allows scientists to understand the interactions and regulatory mechanisms more comprehensively and precisely between different cell types in the tumor microenvironment and provide more accurate and personalized programs for tumor treatment.

## 3 Tumor microenvironment analysis

Tumor microenvironment (TME) refers to the complex network system composed of immune cells, fibroblasts, and other cells, as well as extracellular matrix (ECM) and various signaling molecules, which is closely related to the occurrence, growth, and metastasis of tumor and the generation of drug resistance.

### 3.1 The development of TME

Stephen Paget created the notion of TME more than a century ago, and we will first go through the primary event points mentioned for TME.

**Figure 3:**
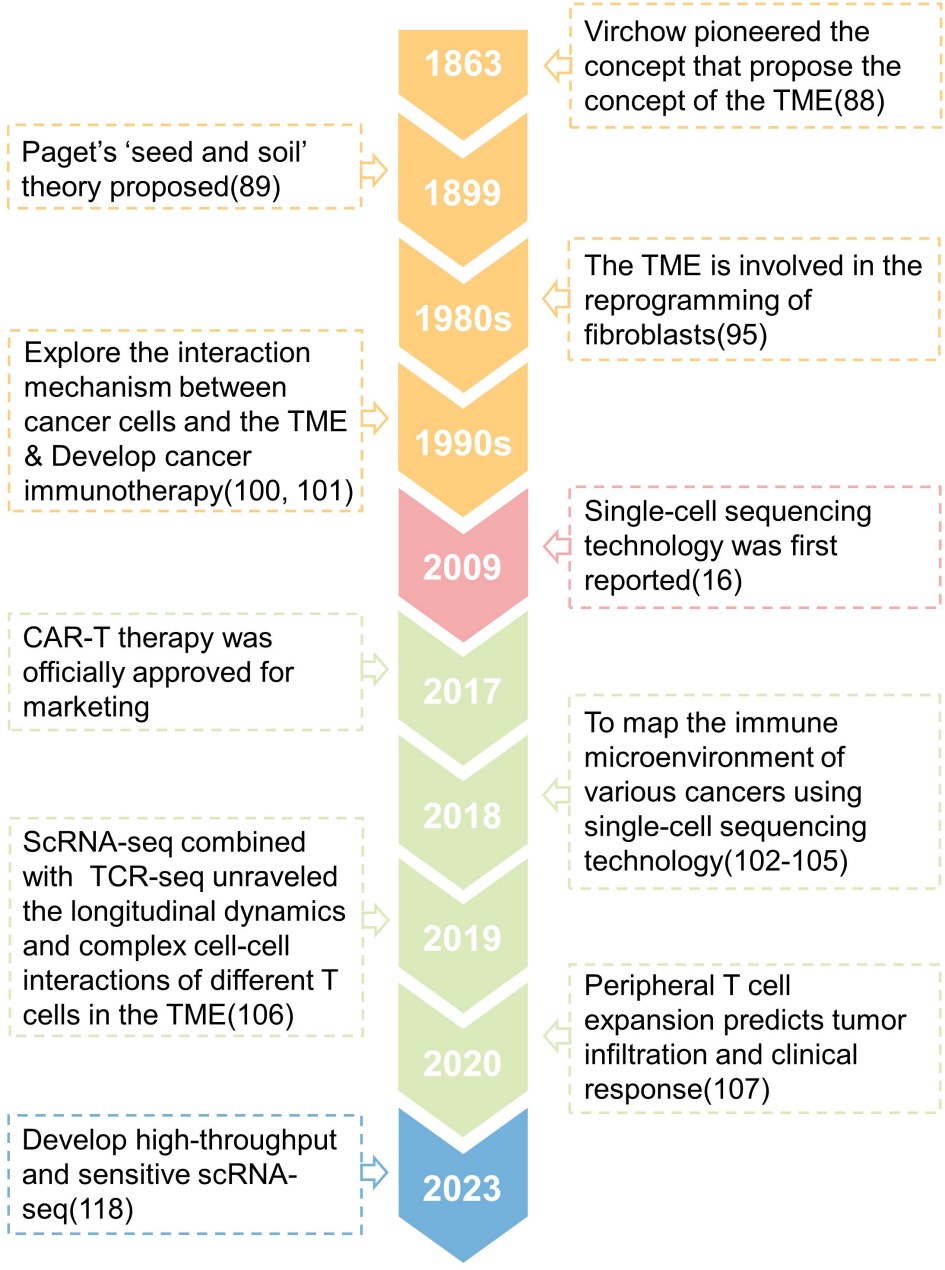
A timeline is presented that shows the key concepts and findings relating to research of the tumor microenvironment (TME).

Around 1863, Rudolph Virchow observed that white blood cells penetrate solid tumors and postulated that inflammation leads to cancer, which was one of the first studies to describe the connection between tumor and its microenvironment (91). Paget’s research shown that tumor metastasis and colonization are affected by the characteristics of the organ, and he proposed the ‘seed and soil’ theory in 1889 (92). This viewpoint is shown to be the foundation of the interaction between the original tumor and its microenvironment, which influences tumor growth and metastasis. Therefore, Paget is regarded as the theoretical forefather of the TME (93–97). People’s research on the TME at the time was just descriptive, and it was unable to analyze its interaction mechanism. Until the 1980s, it was discovered that the signals released by the TME might drive somatic cells to reprogram (98), which considerably enhances tumor cell survival and proliferation (99–102). At this time, researchers began to focus on the interaction mechanism between tumor cells and TME components, and a number of tumor immunotherapies were produced (103, 104). However, as previously stated, the heterogeneity of cells inside tumors results in tumor immunotherapy being effective for only a small number of patients and a significant recurrence rate (4). Since the first release of single-cell sequencing technology in 2009 (16), researchers have utilized it to better define the biological properties of tumor-infiltrating immune cells, which has become critical to understanding tumor immunity and developing tumor immunotherapy. For example, Zhang released two landmark publications in 2018 that used single-cell sequencing technology to construct the STARTRAC system, which detailed described the immune microenvironment of rectal cancer and lung cancer (105, 106). These investigations offer a novel way to patient stratification and will contribute to a better understanding of the functional states and dynamics of T cells in cancer (107, 108). In 2019, an Israeli team discovered the transcriptome heterogeneity and differentiation pathways of melanoma-infiltrating T cells (109), proving that the tumor microenvironment contains a class of immune cell dysfunctional T cells that promote tumor progression. In 2020, the American team additionally demonstrated that peripheral T cell proliferation can predict tumor invasion and clinical response (110), expanding our understanding of the makeup of CD8+T cell subtypes in the local tumor as well as the source of CD8+T cell clonal colonies in the local tumor. Many scientists have continued to use single-cell sequencing technology in recent years to study the TME, revealing the origin, evolution, and dynamics of T cells (111–115), dendritic cells (116, 117), macrophages (118, 119) and other immune cells, also to identify new predictive markers with great potential as a supplement to the existing tumor immunotherapy. However, single-cell sequencing technology based on the second-generation sequencing platform is still constrained by poor accuracy and sensitivity. This year, researchers attempted to use the third-generation sequencing (TGS) platform to perform high-throughput and high-sensitivity full-length single-cell RNA-seq analysis to distinguish transcripts of pseudogenes from those of corresponding parental genes (120), which could help researchers better understand the regulatory mechanisms of genes in single cells (121). Above, we discussed the evolution of the TME research before and after the introduction of single-cell sequencing (**Figure 3**).

### 3.2 Research status of main components of TME

The TME’s makeup is highly complicated, with several components interacting with one another, and comprises the complex networks of cell-cell interactions and cell-non cell interactions that modulate tumor progression and response to therapy (122). Benefit by breakthroughs in single-cell sequencing, knowledge the composition of TME and interpreting tumor tissue heterogeneity will assist increase our knowledge of the underlying processes and create precision medicines for use in clinical trials (123). Here, we cover the research status of each component of TME, as well as the dynamic changes of TME, which is useful in understanding the emergence and progression of cancers, as well as how to adopt precision medicine.

#### 3.2.1 Tumor-associated immune cells

Immune cells play a crucial role in the TME, as they can either suppress or promote tumor growth depending on their subtypes and activation status. Cancer cells usually recruit immune cells to infiltrate around them, forming an immunosuppressive microenvironment conducive to tumor growth (124).

##### 3.2.1.1 Regulatory T cells

T cells are lymphocytes that develop and mature in the thymus after being produced from bone marrow. They are a complicated and irregularly dispersed set of cells. At the moment, the categorization principles and nomenclature are rather muddled, but it is obvious that T cells play a dual function in tumor formation. The regulatory T cells (Tregs) are among the more researched immunosuppressive T cells.

**Figure 4:**
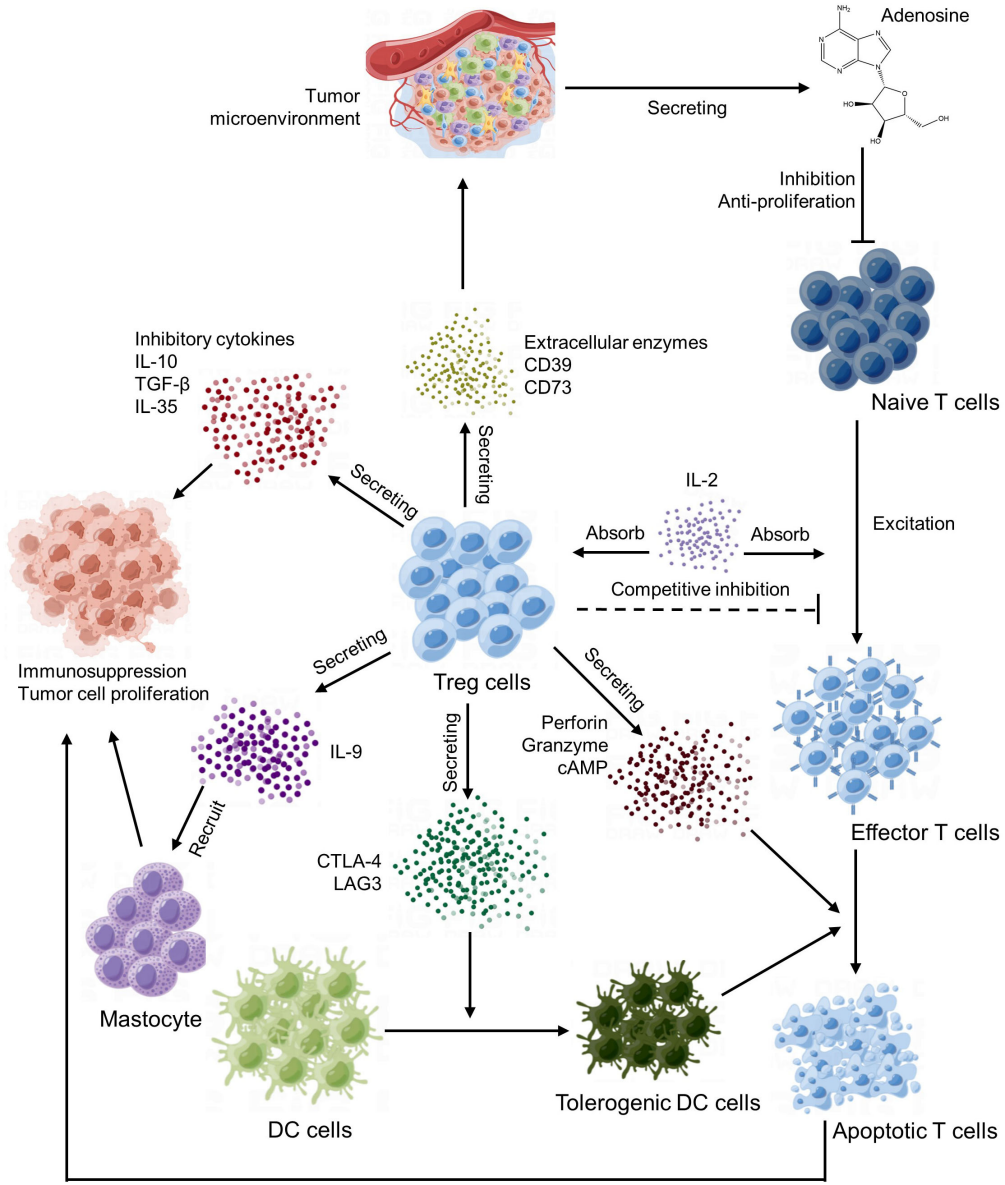
Regulatory T cells (Tregs) regulatory network.

Tregs are important immune regulatory cells that play a crucial role in the tumor microenvironment (125). Tregs maintain the state of immune tolerance mainly by suppressing the autoimmune response and occupy a place in the CD4 T lymphocyte subpopulation. Under normal circumstances, it can prevent autoimmune diseases and limit chronic inflammatory diseases. In the tumor microenvironment, Tregs play an important role in the formation of tumor immune escape and immune tolerance (126). The main way of Tregs action is to produce inhibitory cytokines, inhibit effector T cells and other immune cells and recruit inhibitory immune cells (**Figure 4**).

First of all, Tregs can suppress tumor immune responses by eliminating the activity of autoimmune cells. This mechanism is mainly regulated by signal molecules such as CD25 (IL-2 receptor α‐chain) and CTLA-4 on the cell surface. CTLA-4 mediated suppression of antigen-presenting cells (127) and consumption of CD25 and production of immune inhibitory cytokines and molecules (128).

Additionally, Tregs can also affect the immune response of tumors by directly interfering with and obstructing the function of other immune cells, such as mast cells, dendritic cells and B cells (129). For example, Tregs can reduce antigen presentation and induced immune responses by inhibiting the maturation and activation of dendritic cells (DCs) (130), IL-9 secreted by Tregs cells recruits and activates mast cells, which are essential for cell-dependent peripheral tolerance (131) and granzyme secreted by Tregs cells can kill T cells in the way of cell-mediated perforation, thus inhibiting the function of T cells (132).

In the end, Tregs influence tumor immune response by regulating the metabolism and secretion of cytokines. For instance, Tregs can produce a series of immunosuppressive cytokines, such as TGF-β and IL-10, which can directly inhibit the function of other immune cells and indirectly reduce the intensity of immune response (133).

In summary, Tregs play an important negative regulatory role in the tumor microenvironment, can suppress tumor immune response through a variety of mechanisms, and participate in the formation of tumor immune escape and tolerance. Therefore, some immunotherapy methods targeting Tregs have also become the research hotspot of tumor therapy at present.

##### 3.2.1.2 B cell

B cells tissue and lymphocyte found in the body’s immune system, mostly in bone marrow and lymphoid tissue, and are distinguished by the surface expression of immunoglobulin receptors capable of recognizing and binding antigens. Under normal conditions, after binding to the antigen, B cells will activate the cell via intracellular signaling and differentiate into plasma cells, which secrete a large number of antibodies to recognize and bind to the antigen on the tumor’s surface, assisting the immune system in clearing the tumor cells. Furthermore, it can help other cells carry out anti-tumor effects, such as secreting anti-tumor-associated antigen (TAA) antibodies to NK cells to boost NK cells’ ability to kill tumors, and it can act as a professional antigen presenting cell (APC) (134), presenting antigens to CD4+ helper T cells and CD8+ cells while providing co-stimulatory signals, activating T cells (135, 136). With the advent of single-cell sequencing technology, it was shown that the tumor microenvironment has a tertiary lymphoid structure (TLS), and B cells, as the principal component and originator of TLS, play a role in attracting T cells, DC cells, and other cells (137). It was also shown that substantial B lymphocyte infiltration in malignancies was related with a bad prognosis (138). Further research discovered tumor-associated B cells (Breg) in the tumor microenvironment, and they produced inhibitory molecules including IL-10 and TGF-b to decrease the function of cellular and humoral immunity, favoring tumor formation (139). Furthermore, the immunological complex formed by antibodies generated by specific plasma cells and TAA stimulates the formation of myeloid derived suppressor cells (MDSC), therefore suppressing anti-tumor response (140). It can be shown that B cells play a complicated role in the process of tumor cell proliferation, which affects the diverse orientations of immunotherapy. Further research into how to stimulate anti-tumor B cells and suppress pro-tumor B cells is needed. Using single-cell sequencing tools to describe how to trigger and modify B-cell responses inside TME will aid in the development of therapeutic strategies for cancer immunotherapy.

##### 3.2.1.3 Tumor-associated macrophages

**Figure 5:**
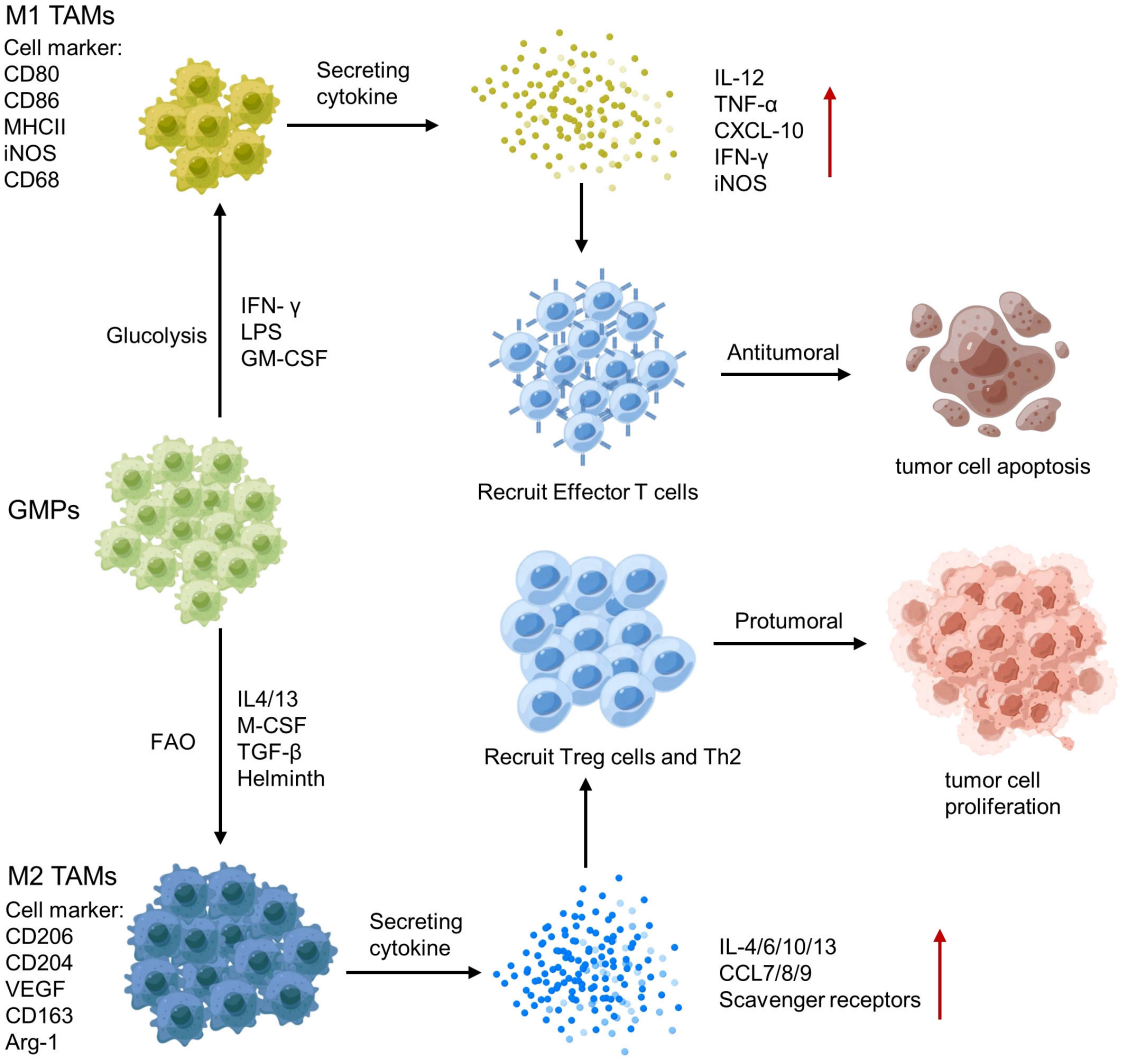
Polarization of tumor-associated macrophages (TAMs) and its regulatory network.

Tumor-associated macrophages (TAMs) are a key component of the TME, and their polarization towards an M1 or M2 phenotype can have different effects on tumor immunity (141). M1 TAMs release pro-inflammatory cytokines, such as IL-12 and TNF-α and produce NO and reactive oxygen species, which activate anti-tumor immune responses (142). In contrast, M2 TAMs secrete anti-inflammatory cytokines, such as IL-10 and TGF-β, growth factors (e.g., vascular endothelial growth factor VEGF) and Matrix metalloproteinases (MMP; e.g., MMP-9 and MMP-2), which promote tumor growth and metastasis (124) (**Figure 5**). Therefore, analyzing the polarization and spatial distribution of TAMs within the TME is crucial for understanding tumor-immune interactions and developing immunotherapeutic strategies.

##### 3.2.1.4 Tumor-associated neutrophile

**Figure 6:**
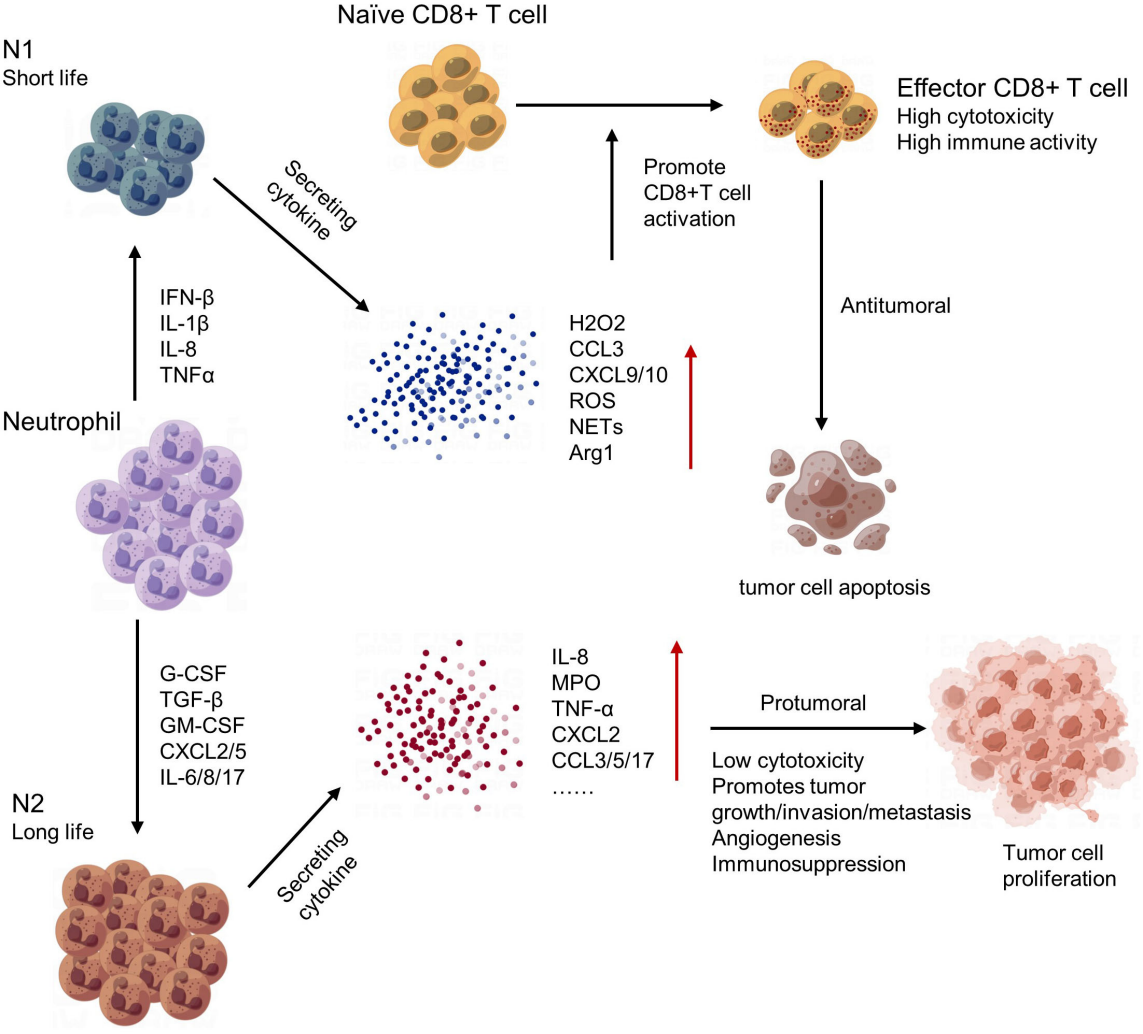
The polarization and regulatory network of tumor-associated neutrophils (TANs).

Neutrophils play an important role in regulating the tumor microenvironment. In early stages of tumorigenesis, neutrophils can exhibit anti-tumor activities by releasing cytotoxic molecules and forming extracellular traps to kill cancer cells. However, in later stages, neutrophils often become pro-tumor and contribute to tumor growth and progression (143). One mechanism by which neutrophils promote tumor growth is through the secretion of growth factors such as vascular endothelial growth factor (VEGF) and matrix metalloproteinases (MMPs), which promote angiogenesis and tissue remodeling. Neutrophils can also create an immunosuppressive environment by inhibiting T cell function and promoting Treg accumulation (144). Moreover, neutrophils can interact with other immune cells and stromal cells in the tumor microenvironment, leading to the recruitment of additional neutrophils and further amplifying their pro-tumor effects (145) (**Figure 6**). In conclusion, neutrophils have complex and dynamic roles in regulating the tumor microenvironment. Targeting neutrophil functions may provide a new approach for cancer therapy.

##### 3.2.1.5 Dendritic cell

Dendritic cells (DC) are white blood cells (146), which Steiman and Cohn identified in 1973. They are the most potent antigen-presenting cells ever known. They are at the heart of initiating, regulating, and sustaining immune responses, and can efficiently extract, process, and present antigens. When dying tumor cells produce danger signals, the released DAMPs encourage DC maturation and migration to draining lymph nodes, where cancer antigens are processed and loaded into human leukocyte antigen (HLA). It also increases co-stimulatory analysis and creates pro-inflammatory cytokines, which efficiently activate early T cells (147). Despite the fact that DC is separated into several subtypes and that there is a clear difference between tissue invading DC in various cancers, high infiltration of DC in tumors generally predicts high survival (148), showing that DC, particularly cDC1s, primarily plays a role in limiting tumor formation and progression. Steinman effectively prolonged his survival after being diagnosed with pancreatic cancer by utilizing DC vaccination (149). Since then, there has been a surge in the development of the DC vaccination, which is currently acknowledged as a relatively successful immunotherapy. Despite the discovery of further clinical uses, many patients continue to fail to react to this medication. When combined with single-cell sequencing technologies, it was shown that some subtypes of DC, such as pDC and cDC2, had both anti-tumor and pro-tumor activities (150). Furthermore, the function of DC will be diminished due to the intricacy of the tumor microenvironment. Reduced DC aggregation at tumor locations, immunosuppressive factor reduction of DC antigen presentation power, and loss of HLA expression capacity, for example, are all ways for tumor cells to elude immunization (151). In a nutshell a good knowledge of the process of immunosuppression might lead to new immunotherapies, combination treatments, and the discovery of biomarkers.

### 3.2.2 Tumor-associated stromal cells

In addition to immune cells, stromal cells such as cancer-associated fibroblasts (CAFs), endothelial cells, and pericytes also contribute to the TME (152). CAFs are the most abundant stromal cell types in the TME. They are recruited and activated by growth factors secreted by immune infiltrating cells and tumor cells, secrete extracellular matrix components and growth factors that support tumor growth and invasion, and participate in recruitment of circulating endothelial progenitor cells as well as immune and metabolic reprogramming of the TME (153).

Endothelial cells form blood vessels that supply nutrients and oxygen to the tumor, and they also play a role in immune cell recruitment and extravasation as well as in the generation and transfer of drug resistance (154).

Pericytes are mural cells that wrap around blood vessels, regulating their stability and function. The interaction between pericytes and endothelial cells is critical for angiogenesis, a hallmark of tumor development (155).

In a word, stromal cells play an important role in tumor growth and metastasis. In the past, the research on tumor microenvironment mainly focused on the composition and function of immune cells. Since the advent of single-cell sequencing technology, the influence of stromal cells on tumor growth has been gradually studied. Studying this role involves studying the interactions between cancer cells and stromal cells to determine the specific mechanisms by which stromal cells influence tumor progression.

### 3.2.3 Tumor-associated non-cellular components

The composition of tumor microenvironment is very complex. In addition to cellular components, non-cellular components are also important components of tumor microenvironment and have an important impact on tumor growth. The main non-cellular components include extracellular matrix (ECM), cytokines and growth factors, micrornas (mirnas), and exosomes (156).

The ECM is a complex network of proteins, polysaccharides and other molecules that can provide support and protection, while also being involved in signaling and regulation between cells. In the tumor microenvironment, tumor cells can change the composition and structure of extracellular matrix, so as to form an environment suitable for their own growth and invasion (157).

Tumor cells and surrounding non-tumor cells can secrete a variety of cytokines and growth factors, including pro-angiogenic factors (e.g., VEGF), inflammatory factors, growth factors (e.g., EGF), etc. These molecules can stimulate the division, proliferation, and invasion of tumor cells, and attract and activate immune cells, affecting the process of tumor development and metastasis (158).

miRNA is a class of short stranded RNA that can be involved in the regulation of various cell biological processes. In the tumor microenvironment, miRNA can be secreted and taken up by tumor cells and non-tumor cells, thus affecting the proliferation, invasion, and metastasis of tumor cells. At the same time, miRNA can also regulate the function of immune cells, affecting tumor antigen presentation and immune surveillance (159).

Exosomes are small vesicles secreted by cells that can carry a variety of molecules, such as proteins, mirnas, and DNA. In the tumor microenvironment, exosomes can promote the proliferation, invasion and metastasis of tumor cells through the transfer of molecules and signals, as well as influence the immune system’s control of the tumor (160).

## 4 Application of single-cell sequencing in cancer TME research

The TME analysis can be performed using different methods, including histology, immunohistochemistry (IHC), flow cytometry, and scRNA-seq. Histology and IHC can provide information on the cellular and morphological features of the TME, but lack the resolution to analyze individual cells’ transcriptomes. Flow cytometry enables the quantification and isolation of specific cell populations within the TME but has limited throughput and sensitivity. The scRNA-seq allows the profiling of individual cells within the TME, providing insights into the cellular composition, gene expression patterns, and functional interactions.

**Figure 7:**
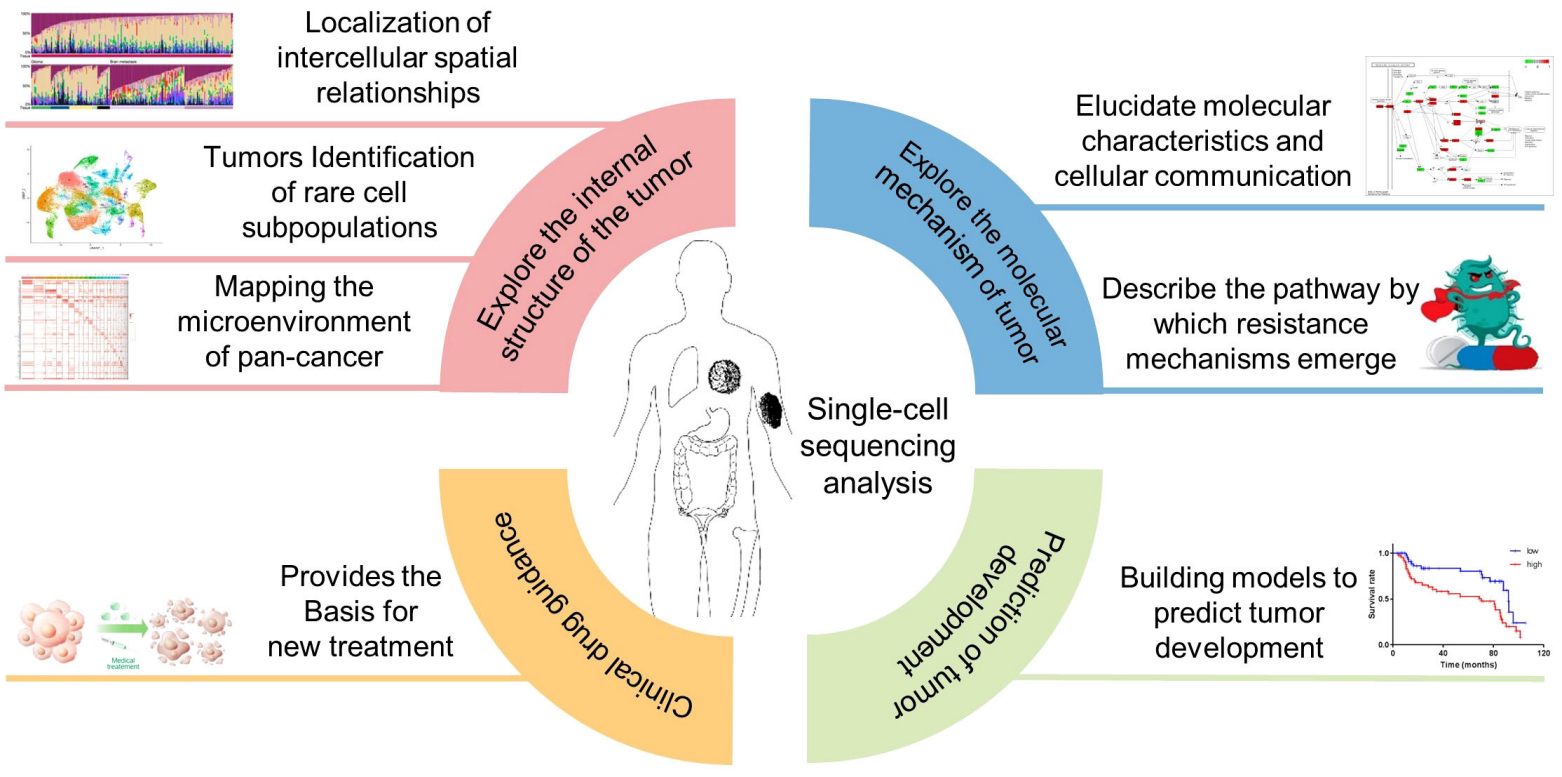
Application of single-cell sequencing in cancer the TME research. Inclusion four main aspects, exploring the internal structure of the tumor, explore the molecular mechanism of tumor, Clinical drug guidance, Prediction of tumor development.

The combination of single-cell sequencing and the TME analysis provides a powerful tool for investigating the molecular mechanisms underlying tumor progression, response to therapy, and immune evasion. Recent studies have shown the potential of single-cell sequencing and the TME analysis in various cancer types, including gastric cancer (161), ovarian cancer (162), multiple myeloma (163), melanoma (164), lung cancer (165), breast cancer (166) (**Figure 7**).

With around 22,000 new cases and 17,900 fatalities each year (167), lung cancer is the most prevalent malignant tumor among malignancies and the leading cause of cancer-related mortality. Breast cancer also has the highest worldwide new incidence rate of female cancer and the second highest fatality rate (168). As a result, the present global study on lung cancer and breast cancer is also more comprehensive. This section describes in detail the application of single-cell sequencing technology in the study of lung and breast cancer tumor microenvironments and how to guide precision medicine, as well as a brief description of the more advanced application of this technology in the study of melanoma, ovarian cancer, and multiple myeloma.

### 4.1 Application of single-cell sequencing in the TME of lung cancer research

Lung cancer is the leading cause of death in humans (169). Despite recent breakthroughs in targeted treatment and immunotherapy (170), patients continue to face medication resistance and inadequate immunotherapy (171). Single-cell investigations can assist in a better understanding of heterogeneity *in vivo* and across patients, increase the resolution of cell difference studies, and help guide therapy (172). Ashley Maynard et al. (173) employed RNA-seq to evaluate lung cancer samples at various stages of treatment, demonstrating the rich and dynamic tumor ecology of lung tumors during treatment. Fengying Wu et al. (165) assembled a significant sample of advanced patients for single-cell analysis, providing a very detailed picture of the TME and its heterogeneity for advanced lung cancer research. Nevertheless, alterations in the TME can influence cancer cell metastasis. As a result, through single-cell transcriptome analysis of metastatic lung adenocarcinoma, Nayoung Kim et al. (174) discovered the cancer cell subtypes that dominate metastasis and mapped the changes in tumor microenvironment and immune cell dynamics during metastasis. Baolin Liu et al. (175) analyzed 47 samples of non-small cell lung cancer treated with PD-1 combination chemotherapy using the scRNA-seq, scTCR-seq, and bulk TCR-seq platforms to comprehensively explore the dynamic alterations of T cells before and after anti-PD-1 combined therapy. ​Molecular differences between patients with different therapeutic effects were analyzed, while the concept of clonal revival was proposed to provide new insights into the underlying mechanisms of PD-1 therapy. Although tumor immunological microenvironment has been characterized in great detail (176–179), the majority of them rely on histopathological tumor subtype classification and acquaint scarcely about the spatial status of single-cell phenotypes within stratified subpopulations (180). As a result, Mark Sorin et al. used an imaging mass spectrometer, single-cell sequencing, and other technologies to analyze the spatial immune lineage and activation status of tumors in 5 samples with histological patterns in order to better predict patients’ postoperative progress using small biopsy samples, demonstrating the use of high spatial resolution in single-cell analysis. It also stresses the importance of artificial intelligence in the advancement of tumor microenvironment research.

Because the TME, therefore, play an important role in the development and treatment of lung cancer (181, 182), so understanding lung tumor TME on time and space diversity is crucial, although the study of immune cells in the TME now has a certain depth, but for the TME another important composition of stromal cells and their secretions research limited (183). Therefore, future studies may focus on characterizing the TME composition and microenvironment remodeling, as well as the network and functional crosstalk between immune cells and stromal cells in the TME through single-cell sequencing technology with high resolution and high fidelity, so as to provide more strategies for immunotherapy.

### 4.2 Application of single-cell sequencing in the TME of breast cancer research

Breast cancer is the most common malignancy in women globally (184), and it is characterized by a highly diverse malignant tumor that develops in breast epithelial tissue (185). In 2018, Elham Azizi et al. (107), constructed the atlas of breast cancer immune cells through single-cell sequencing technologies including scRNA-seq and scTCR/BCR-seq, revealing the diversity of immune cells and extensively characterizing the heterogeneity of immune cells in breast cancer tissues. The effect of tumor microenvironment on immune cell phenotype in breast cancer subtypes was evaluated. This will help researchers better grasp the functional processes by which immune cells promote and hinder tumor growth. By 2021, Li Hu et al. (186)’s work, which revealed the cell source and evolutionary path of breast cancer in BRCA1 gene mutation carriers by combining scRNA-seq with Bulk RNA-seq and WES. By using scRNA-seq on normal tissue, cancer tissue, and metastatic tissue, Bhupinder Pal et al. (187) created the most thorough transcriptional map of human breast tissue and investigated the varied heterogeneity of breast cancer. The results of this study not only contribute to people’s understanding of the mechanism of breast cancer, but also understanding of the mechanism of breast cancer. It also helps to comprehend how breast cancer is advanced and disseminated by cells in the tumor microenvironment. Single-cell and spatially resolved transcriptome maps of human breast cancer were created in the study by Sunny Zwu et al. (188) to expose the heterogeneity of recurrent tumor cells and further the development of ersonalized treatment for breast cancer. Compared to other breast cancer types, triple negative breast cancer (TNBC) is more deadly and aggressive (189), and it responds poorly to traditional breast cancer treatments (190). Thus, using a variety of single-cell sequencing technologies, including scRNA-seq, scATAC-seq, and scTCR-seq, Yuanyuan Zhang et al. (191) revealed the dynamic characteristics of immune cells in patients treated with paclitaxel and attillizumab as well as the changes of immune characteristics related to patient response. It provides a reliable basis for further understanding the immune characteristics of TNBC patients and the mechanism of action of immunotherapy combined with chemotherapy. Furthermore, Siyu Guo et al. (192) employed scRNA-seq data to examine T-cell heterogeneity in TNBC tumor microenvironment, and created and validated the risk models related to T-cell marker genes and prognosis, which are useful in predicting TNBC treatment response and prognosis.

Obviously, single-cell sequencing technology facilitates the analysis of the breast cancer microenvironment, providing novel insights into the phenotype and functional characteristics of tumor invasion and cellular diversity. It unveils new anti-tumor responses in cells, identifies potential therapeutic targets, and promotes the advancement of personalized treatment.

### 4.3 Application of single-cell sequencing in the TME of other cancer research

Junbin Qian et al. (193), used scRNA-seq and cell surface protein detection technology on samples from lung cancer, colon cancer, ovarian cancer, and breast cancer to create a pan-cancer cell map of the heterogeneity of the TME, which was then used to identify melanoma patients treated with checkpoint immunotherapy. The emergence of this pan-cancer blueprint for predicting the response of different naive CD4+T cell phenotypes to immune checkpoint therapy can aid researchers in understanding the pathways by which various resistance mechanisms emerge after treatment, which is a significant step forward in the development of new cancer drugs and mechanistic research.

Melanoma has long been a serious problem for cancer research and public health due to its high heterogeneity and variability (194). Researchers used scRNA-seq to show that melanoma cells can have both proliferative (or melanocyte) and mesenchymal (or invasive) transcriptional cell states (195). However, clinical sample investigations indicate that melanomas may contain more than just these two cell types (196). Therefore, Karras P et al. (197), conducted a collaborative study using spatial transcriptome techniques such as scRNA-seq, lineage tracking, and Stereo-seq to investigate potential melanoma cell subsets, determine their growth structure and spatial environment, and create a high-resolution and spatially resolved map of the mouse melanoma ecosystem. The cause of phenotypic diversity and tumor growth has been discovered. At the same time, their Met-Track mouse model provides a foundation for additional research into the exterior and systemic mechanisms that drive primary tumor growth and spread.

The tumor microenvironment successfully gastric cancer has been successfully decoded using scRNA-seq, and its possible tumor biological mechanism has been examined and revealed (198). However, immunotherapy targeting PD-1 and CTLA4 antibodies, which are extremely effective in the treatment of other malignancies, is not satisfactory in the treatment of gastric cancer (199). Keyong Sun et al. (161), provided a very thorough transcriptome map of single-cell gastric cancer, providing precise and sophisticated classification of immune subsets, stromal subsets, and epithelial subsets using scRNA-seq analysis and TCR/BCR library profiles. Spatial transcriptomics and scATAC-seq were employed to further elucidate the important molecular features and intercellular communication of these interacting cell types indicated by CellPhoneDB. The most visible characteristic in the TME is the significant remodeling of cellular components, with inhibitory Tregs, TASCs, TAMs, Tc17 and CD8 + deficient T cells enriched in the tumor and mast cells, endocrine and follicular regulatory T cells enriched outside the tumor. TASCs, M, APOE, and LAMP3 + dc, which are cell-cell interaction mediators, can promote the formation of immunosuppressive microenvironment and tumor formation, elucidating the relationship between cell subsets in tumor microenvironment and tumor progression and providing some promising clues for tumor therapy.

Congcong Yan et al. (162), used combined single-cell sequencing and tissue sequencing to analyze the dynamic heterogeneity of the tumor microenvironment in ovarian cancer, and they systematically described the relationship between the pattern of macrophage activation and prognosis and treatment response. This research investigates the immunological microenvironment from tissue data, turns to single-cells for verification and complementation, and finally returns to the idea of tissue guidance for typing and prediction of clinical outcomes that is the main idea of this article.

Minghao Dang et al. (163), used scBCR-seq to investigate the heterogeneity of clonal plasma cells in patients with multiple myeloma precursor disease, the early genomic drivers of myeloma malignant transformation, and the differences in the transcriptome profile and tumor microenvironment between hyperdiploidy and nonhyperdiploid genotypes. Furthermore, the study deduced diverse clonal evolution pathways from precursor disorders to myeloma. This study advances our understanding of the course of myeloma precursor illness and gives important insights into patient risk classification, biomarker discovery, and potential treatment applications.

### 4.4 Summary of application of single cell sequencing technology

Indeed, single-cell sequencing technology has affected many fields of cancer research, boosting understanding of the TME, intra tumor heterogeneity, metastasis, and therapy resistance, as well as effectiveness prediction. For example, single-cell transcriptome analysis was used to confirm the relationship between macrophage functional status and response to chemotherapy, and existing data was used to develop algorithms to predict patient response to chemotherapy and immunotherapy based on the degree of infiltration of different subtypes of macrophages, with the goal of predicting clinical outcomes (162). The tumor microenvironment is examined using a mix of single-cell transcriptome sequencing and spatial transcriptome to identify novel cells or important components involved in tumor formation. For example, because the content of brain fibroblasts was so low in the past, it was assumed that there were no tumor-associated fibroblasts (CAF) in glioblastoma, hence therapy of glioblastoma (GBM) was restricted to the tumor cells alone, resulting in a poor therapeutic effect (200). Through single-cell transcriptome sequencing and spatial transcriptome combination, Saket Jain et al. (201) demonstrated the existence of fibrocytes that support tumor development in GBM, which enhanced our understanding of GBM and contributed to further study and treatment of GBM. Because tumor cells are highly mutable, they may swiftly adapt to environmental changes caused by medications and rapidly develop. To administer targeted medications to such tumors, it is crucial to first study their distinct differentiation processes. Sergi Beneyto-Calabuig et al. (202) discovered clonally resolved single-cell multi-omics identifies reveals a differentiation landscape that mimics its healthy counterpart and may determine biology and therapy response in AML. Overall, the application of single-cell sequencing technology in cancer research at this stage is primarily to analyze the TME, and then to discover the rule for early cancer detection, non-invasive detection, improve clinical diagnosis and risk stratification, and discover new drug targets.

## 5 Summary of challenges of single-cell sequencing technology

Although the fast growth of single-cell sequencing technology has increased the application of its study in recent years, delivering revolutionary new technologies for biological research, there are still certain issues with the method itself. Sample collection, processing, and sorting in the data construction stage require a high level of skill and experience, as well as the use of special equipment, which has high requirements for experimental personnel and experimental platforms. At the same time, this technology has a certain demand for sample quality, so this technology is expensive and not civilian. Furthermore, the present separation technique might produce unanticipated cell stress during the process of creating library sequencing, affecting the behavior, vitality, and features of the cells, leaving them in an unnatural condition that will impair later analysis. Post-single cell sequencing analysis requires data preprocessing and optimization in the data integration stage to deal with a large number of complex data generated by sequencing, and different sequencing platforms have been developed based on different database construction methods, with varying levels of resolution, coverage, and fidelity. Additionally, while each cell is assigned a unique identifier during the sequencing process, it is impractical to evaluate such a huge number of cells separately during the analysis, hence dimension reduction is necessary, which is aggregated a collection of similar cells together for analysis, significant differences may be lost throughout the process of integrated data analysis in order to dimensionality reduction and balance the resolution. Up to now, researchers are still being made to reduce the bar, for example, Bio-Rad and Illumina are working on a solution to streamline the entire single-cell sequencing workflow in order to achieve high throughput, minimal time, and a cheap cost of single cell sequencing. In terms of cell type identification, because the cell state is dynamic, the data obtained from each single cell sequencing will be different, and researchers have their own naming methods, resulting in the current naming of cell subtypes being very confusing, and there is no uniform naming standard, which will cause significant problems with your understanding of TME. As well as the marker employed for cell subtype identification is disunity, and how to verify scarce and new-found cell subtypes is also a challenging task to tackle. On a technical level, there is currently a shortage of methods for combined sequencing and analysis, particularly for histone modification or transcription factor joint analysis, which is equally important at the molecular regulatory network level. To summarize, while single-cell sequencing technology was selected Technology of the Year in 2013, there is still much space for advancement.

## 6 Prospect of single-cell technologies in tumor research

In conclusion, single-cell sequencing has helped to break down research barriers and has now transformed many areas of cancer research, describing the diversity of cells in the TME niche and providing a more complete blueprint for tumor heterogeneity, TME composition, tracer cell formation, and functional interactions. Integration with multi-omics data, such as DNA sequencing, RNA sequencing, and protein analysis, can help us understand tumorigenesis, immunotherapy response, and treatment resistance even more. The challenges mentioned above will be well solved as technology advances, lowering the threshold for single-cell sequencing even further, which will better promote TME research, explore the heterogeneity of TME before and after treatment, aid in tumor classification, develop personalized cancer early screening and late treatment, pave the way for precision oncology, and is expected to have a greater clinical impact. Single-cell sequencing will touch more areas of cancer care and become a routine tool in hospitals, much as next-generation sequencing (NGS) did for current oncology research. We anticipate that the use of single-cell sequencing technologies in cancer care over the next decade will result in major breakthroughs in cancer diagnosis and therapy.

## Author contributions

SC: Writing – original draft, Writing – review & editing. ZZ: Writing – review & editing. YL: Writing – review & editing. DY: Writing – review & editing. GC: Writing – review & editing.
