# Corrigendum: Application of single-cell sequencing to the research of tumor microenvironment

**DOI:** 10.3389/fimmu.2023.1345222

**Published:** 2023-12-05

**Authors:** Sijie Chen, Zhiqing Zhou, Yu Li, Yuhui Du, Guoan Chen

**Affiliations:** Department of Human Cell Biology and Genetics, Joint Laboratory of Guangdong-Hong Kong Universities for Vascular Homeostasis and Diseases, School of Medicine, Southern University of Science and Technology, Shenzhen, China

**Keywords:** single-cell sequencing, tumor microenvironment, clinical applications, precision medicine, immunological therapy

In the published article, there was an error in affiliation 1. Instead of “**Department of Human Cell Biology and Genetics, Joint Laboratory of Guangdong-Hong Kong Universities for Vascular Homeostasis and Diseases, School of Medicine, Southern University of Scienece and Technology, Shenzhen, China**”, it should be “**Department of Human Cell Biology and Genetics, Joint Laboratory of Guangdong-Hong Kong Universities for Vascular Homeostasis and Diseases, School of Medicine, Southern University of Science and Technology, Shenzhen, China**”.

The authors apologize for this error and state that this does not change the scientific conclusions of the article in any way. The original article has been updated.

